# Social Capital and Risk of Concurrent Sexual Partners Among African Americans in Jackson, Mississippi

**DOI:** 10.1007/s10461-019-02770-8

**Published:** 2019-12-28

**Authors:** Yusuf Ransome, Karlene Cunningham, Miguel Paredes, Leandro Mena, Cassandra Sutten-Coats, Philip Chan, Dantrell Simmons, Tiara C. Willie, Amy Nunn

**Affiliations:** 1 Department of Social and Behavioral Sciences, Yale University School of Public Health, 60 College Street, LEPH, New Haven, CT 06510 USA; 2 Department of Psychiatry and Behavioral Medicine, Brody School of Medicine, East Carolina University, Greenville, NC USA; 3 Department of Epidemiology of Microbial Diseases, Yale University School of Public Health, New Haven, CT USA; 4 Department of Medicine and Population Health Science, University of Mississippi Medical Center, Jackson, MS USA; 5 Department of Behavioral and Social Health Science, Brown University School of Public Health, Providence, RI USA; 6 Rhode Island Public Health Institute, Providence, RI USA; 7 Department of Medicine, Brown University, Providence, RI USA; 8 Division of Infectious Diseases, The Miriam Hospital, Providence, RI USA; 9 Department of Health and Human Services, Substance Abuse and Mental Health Service Administration, Washington, DC USA

**Keywords:** HIV, African american, Social capital, South, Mississippi

## Abstract

Concurrent sexual partnerships (i.e., relationships that overlap in time) contribute to higher HIV acquisition risk. Social capital, defined as resources and connections available to individuals is hypothesized to reduce sexual HIV risk behavior, including sexual concurrency. Additionally, we do not know whether any association between social capital and sexual concurrency is moderated by gender. Multivariable logistic regression tested the association between social capital and sexual concurrency and effect modification by gender. Among 1445 African Americans presenting for care at an urban STI clinic in Jackson, Mississippi, mean social capital was 2.85 (range 1–5), mean age was 25 (SD = 6), and 62% were women. Sexual concurrency in the current year was lower for women compared to men (45% vs. 55%, *χ*^2^(df = 1) = 11.07, *p* = .001). Higher social capital was associated with lower adjusted odds of sexual concurrency for women compared to men (adjusted Odds Ratio [aOR] = 0.62 (95% CI 0.39–0.97), *p* = 0.034), controlling for sociodemographic and psychosocial covariates. Interventions that add social capital components may be important for lowering sexual risk among African Americans in Mississippi.

## Introduction

African Americans remain the racial group with the highest prevalence and incidence of HIV infection in the United States (U.S), especially in the south [1]. The southern region, where more than 50% of African Americans reside, is the region with the highest lifetime HIV risk [2]. Jackson, Mississippi (MS), the setting for this study, ranks 6th for new HIV diagnosis and 1st for late HIV diagnosis (i.e., HIV concurrently diagnosed with AIDS) among 95 metropolitan areas in the US [3]. Disparities by race and gender are pronounced. Within Jackson, MS, in 2017, the rate of infection per 100,000 was 62 for African American men compared to 7 for white men, 15 for African American women, and 2 for white women [4].

Racial and ethnic disparities in HIV are not wholly explained by differences in sexual or drug use risk behaviors but rather, attributed to other factors such as delays in testing and accessing HIV prevention, differences in sociodemographic factors of sexual partners (e.g., age and gender), higher HIV prevalence in sexual networks, assortative sexual mixing, and concurrent sexual partnerships [5–8]. Concurrent sexual partnerships (i.e., sexual relationships that overlap in time), is one type of sexual network characteristic that can raise HIV acquisition risks [9] and the magnitude of risk is hypothesized to vary by local epidemiologic context [10]. In some American cities with a high proportion of African Americans, structural factors, including incarceration and poor economic prospects increase the flow of people that leave while also creating imbalances in male to female sex ratios among those who remain [11, 12]. Jackson, MS is the 3rd highest racially segregated city in the U.S [13], and the overall population of MS has been declining, primarily due to domestic outmigration [14].

Previous studies have identified high rates of concurrent sexual partnerships in Mississippi [15, 16]. Therefore, understanding risk factors for concurrent sexual partnerships may inform interventions that reduce self-reported HIV risk behaviors.

Social capital is a multidimensional construct that is operationalized typically within two broad approaches: cognitive and structural [17]. The construct is more frequently conceptualized as a property of the community/neighborhood [18], while others view it as a property of individuals or networks [19]. The cognitive approach, also sometimes referred to as social cohesion, emphasizes perceptions of trust, sharing, and reciprocity. In contrast, the structural approach emphasizes social networks, civic engagement, participation in organiztions, social control, and other group-level properties. There is some debate whether social cohesion is an antecedent to social capital [20]. Others find utility in seeing both as one broad construct [18], encompassing multiple forms, and connected to social embeddedness [21]. Thus, we define social capital broadly as collective resources available to individuals based on social connections [18, 19]. In this study, perceived neighborhood social capital was operationalized at the individual level. This means that individuals reported their perceptions, but the data were not aggregated to the neighborhood level because geographic identifiers were not available. Social capital has been put forth in many theoretical models as a determinant that influences self-reported HIV risk and transmission [22, 23].

Although seen as a broad concept, cognitive-based indicators often used to operationalize social capital in health-related studies have varied tremendously, including in studying HIV/AIDS [24–26]. Some mechanisms are related to the acquisition of social capital at the individual level as well as facilitating links to health behaviors. Some mechanisms hypothesized to link social capital at the individual level to health behaviors include diffusion of information and psychosocial processes that improve coping, self-esteem, and respect. Those mechanisms either have direct effects or buffer the effects of other determinants such as poverty, depression, and excessive alcohol use [18, 27–29]. Behavioral and psychological factors such as excessive alcohol use, depression, and self-reported HIV risk may also vary by gender and thus are important to examine as main variables rather than confounders. African American women, compared to men, have lower rates of drinking and alcohol abuse [30]. While depressive symptoms may be manifested and thus diagnosed differently among African Americans [31], the diagnosed prevalence is higher among women compared to men [32]. Finally, although self-reported HIV risk is complex in that it depends on relationship status and other contextual factors such as HIV prevalence [33], African American women compared to men identify more frequently with lower self-reported HIV risk [34, 35].

There is compelling evidence that social capital is associated with HIV-related risk and protective factors such as condom use, number of sexual partners, and HIV testing [26]. It is unclear, however, whether social capital is associated with sexual concurrency, which is the first question we investigate in this study. Broadly, mechanisms hypothesized to link social capital to lower self-reported HIV risk include higher access to material resources, which can leverage negotiating safer sexual practices such as using condoms and reducing the number of overlapping sexual partners [36, 37]. It is also possible that higher social capital could increase HIV transmission risk if higher perceived trust in one’s community and residens facilitates greater opportunities for casual sexual relationships with multiple people [38].

Based on theory and empirical evidence, it is plausible that gender may modify the relationship between social capital at the individual level and self-reported HIV risk behaviors, such as sexual concurrency, which is the second question we investigate in this study. The theory of gender and power posits that there are gendered relationships between men and women [39] that could influence risk. The division of labor can produce gender differences in self-reported HIV risk, particularly in the south, because women are posited to more heavily rely on their social networks for support [40]. For instance, African American women in Mississippi earn fifty-five cents for every dollar earned by a white man [41] and the average cost of child-care exceeds 20% of women’s median annual earnings. Women compared to men also tend to have higher levels of family responsibilities (e.g., raising children) and consequently may have limited time to engage in social activities that accumulate social capital as well as financial resources [42].

There are also gendered relationships within cultural norms that structure women’s perceptions of expectations of sexual roles and intimate relationships as well as how society expects women to behave [43, 44]. Also, individual and societal perceptions and expectations of sexual roles, structural conditions such as incarceration differentially affect social capital levels between African American men and women. High incarceration rates of African American men in the U.S [45]. shrink the pool of potential partners [46] and result in higher female-to-male sex ratios. These conditions create higher likelihoods that African American women end up or stay in non-monogamous and overlapping sexual relationships with their male partners [40]. Other work has shown that prior incarceration is associated with an increased rate of lifetime sexual partnerships for men [47].

There is compelling empirical evidence of gender differences in the association between social capital and HIV diagnosis and sexual risk behavior [48]. However, the evidence is mixed about the directions of associations and no studies have examined sexual concurrency. One study in Eastern Zimbabwe showed that social capital was associated with lower HIV incidence, yet the magnitude of the association was larger for women compared to men. Women were able to leverage their social capital to adopt safer sexual practices [49]. One study among adults in South Africa found that cognitive social capital (e.g., perceived reciprocity and support) was significantly associated with higher odds of consistent condom use and condom use at last sex for men but not for women [50]. That study did not provide sufficient explanations for the gendered patterns of those associations.

Understanding the role of social capital in having concurrent sexual partnerships (hereafter, sexual concurrency), as one HIV acquisition risk factor, may be necessary to reduce racial disparities, especially among women, in the Mississippi and across the south.

In the present study, we examined the association between social capital and sexual concurrency and then tested whether gender modified that association. As a second objective, we tested the hypothesis that psychosocial and behavioral factors: excessive alcohol use, depressive symptoms, and self-reported HIV risk attenuate any gender differences found.

## Methods

### Study Design and Sample

Data were drawn from a cross-sectional study of 1542 individuals who presented for care at a publicly funded STI clinic in Jackson, Mississippi. Recruitment and data collection procedures have been previously described Nunn et al. [51]. Briefly, individuals presenting for care between January and June 2011 were offered participation in the study; 93% of individuals accepted. Individuals were eligible to participate if they met the following criteria: (1) at least 18 years of age, (2) presenting for STI and HIV screening, (3) willing to complete a 30 min computerized behavioral survey, and (4) spoke English. Participants did not receive compensation for their participation, and all provided informed consent before completing the self-administered computerized survey. The study was approved by the institutional review boards at the University of Mississippi Medical Center, the Mississippi State Department of Health, and The Miriam Hospital in Providence, Rhode Island. We restricted this sample to African Americans (n = 1445).

### Measures

#### Social Capital

The computerized survey solicited information regarding sociodemographic characteristics, substance use, sexual behavior history, access to medical care, as well as structural factors. A social cohesion-based approach [18] was used to operationalize perceived neighborhood social capital, which was assessed using a validated scale [52]. Participants were asked to rate the following statements on a scale of 1 to 5 (1 = strongly disagree and 5 = strongly agree): (1) “people around here are willing to help their neighbors”, (2) “people in my neighborhood can be trusted”, (3) “people in my neighborhood generally get along with each other”, (4) “people in my neighborhood generally share the same values”, and (5) “I live in a close-knit neighborhood”. Consistent with prior work [52], a z-scored summary index variable with mean of 0 and SD of 1 was created, rather than analyze individual items. This index variable was created through Structural Equation Modeling (SEM) confirmatory factor analysis techniques [53] using STATA v14.1 software [54]. Although the questions ask about their neighborhood and neighbors, the data are all at the individual level.

#### Concurrency

To determine sexual concurrency, the following information was solicited from participants: “Please list your three most recent sexual partners in the past 6 months.” Sex, in the context of the survey, was defined as vaginal, anal or oral intercourse. Patients were then asked the following binary (yes/no) question: “During the time period you were having sex with PARTNER INITIALS, did you also have other sexual partners?” If participants responded “yes”, they were coded as engaged in a concurrent partnership for either the past or current year.

#### Covariates

In our analyses, we included the following socioeconomic variables: age, gender (men vs. women), sexual orientation (heterosexual, homosexual, bisexual), marital status (single, married or in a common-law union, divorced or other), highest level of education obtained (high school or less, some college, college degree or higher), monthly gross income (< $500, $501–$1500, $1501–$3000, > $3000), employment (full or part-time, unemployed and looking for work, unemployed and not looking for work), and receipt of public assistance (yes vs. no).

#### Psychosocial and Behavioral Variables

In addition, to examine whether psychosocial and behavioral factors can attenuate gender differences in social capital and sexual concurrency, the following variables were examined: excessive alcohol use operationalized through the Alcohol Use Disorders Identification Test (AUDIT)-C cutoff (on a scale of 1–12 based on 10 AUDIT-C questions where ≥ 4 for men or ≥ 3 for women was considered hazardous use), self-reported depression (In the past 30 days, have you felt downhearted or depressed for more than a day? yes vs. no), and self-reported HIV risk (How would you rate your own HIV risk? not at risk, low, moderate, high).

#### Statistical Analyses

We first examined the distributional properties of each variable. To assess the relationship between social capital and sexual concurrency in our analytic sample, we used multivariable logistic regression, adjusting for age, gender, sexual orientation, marital status, education, income, employment, and public assistance (Model 1).

In order to examine effect modification (hereafter, interaction) between gender and continuously coded social capital in our sample, we created an interaction term between gender*social capital and entered it into the model, adjusting for covariates. We tested for differences on the multiplicative scale between men and women using the Adjusted Wald Test and confirmed the results by performing interaction contrasts of gender differences on the probability scale (margins), and on the additive scale since interactions may be present on one scale but not another [55, 56]. We then calculated the relative excess risk due to the interaction (RERI) and test the significance of the RERI using STATA code provided by VanderWeele and Knol 2014 [57]. Social capital was coded into a binary variable to calculate the RERI since values have to be chosen even from continuously scored variables. We created a binary variable (− 2/0 classified as low and anything higher classified as high).

To accomplish our secondary objective, whether psychosocial and behavioral factors can attenuate gender differences in social capital and concurrency, an additional model was created (Model 2) in which we subsequently adjusted for excessive alcohol use, self-reported depression, and self-reported HIV risk. To improve specification of the model, we entered an interaction term between AUDIT-C hazardous alcohol use and self-reported HIV risk because prior work showed strong causal associations in the context of sexual risk [58]. All variables that were significant at *p* < 0.10 in bivariable analyses were included in both models. We reported two-sided *p-*values. We assessed the significance of the interactions at *p* < 0.05. When significant interactions were found, we plot the results using the margins command based on the adjusted Model 2.

## Results

Table 1 shows the sociodemographic characteristics of the analytic sample. The average age was 24.7 years, approximately 61.5% of the participants were men, single (87.3%) and heterosexual (89.6%). Over forty percent (42%) of the sample had a high school or less education and a little over half (53.2%) of the participants reported being employed full or part-time with almost sixty percent (58.7%) receiving public assistance. A total of 482 participants (34.8%) self-reported depression. About 48% met criteria for hazardous alcohol use, while 48.8% had some self-reported HIV risk. Fifty-five percent of men and 45% of women reported having concurrent sexual relationships in the current year. The average social capital score for the whole analytic sample was 2.84 (with 1 being the lowest score and 5 being the highest), and there were no significant differences in mean social capital between men and women (results not displayed).

**Table 1:** Descriptive characteristics of the study sample

N = 1445	Mean (SD) or N (%)
Social capital (range 1 low to 5 high)	2.84 (0.82)
Gender	
Men	555 (38.51)
Women	886 (61.49)
Age (continuous)	24.70 (6.14)
Sexual orientation	
Heterosexual (ref)	1,282 (89.59)
Lesbian	28 (1.96)
Gay	42 (2.94)
Bisexual	79 (5.52)
Marital status	
Single (ref)	1,261 (87.33)
Married/common-law	112 (7.76)
Divorced/other	71 (4.92)
Degree	
High school or less	605 (41.96)
Some college	637 (44.17)
College graduate and higher	200 (13.87)
Income	
< $500	446 (31.45)
$501–$1500	525 (37.02)
$1501–$3000	276 (19.46)
> $3000	171 (12.06)
Employment	
Full time or part-time	764 (53.24)
Unemployed looking for work	530 (36.93)
Unemployed not looking for work	141 (9.83)
On public assistance	
No (ref)	593 (41.30)
Yes	843 (58.70)
Excessive alcohol use (AUDIT-C) * ≥ 4 for Men and ≥ 3 Women	
No (ref)	747 (51.8)
Yes	694 (48.2)
Self-reported depression	
No (ref)	905 (65.25)
Yes	482 (34.75)
Self-reported HIV risk	
Not at risk (ref)	709 (51.23)
Low	472 (34.10)
Moderate	155 (11.20)
High	48 (03.47)
Sexual concurrency (past year), men	236 (52.10)
Sexual concurrency (past year), women	336 (42.87)
Sexual concurrency (current year), men	249 (54.97)
Sexual concurrency (current year), women	354 (45.15)

**Fig. 1:**
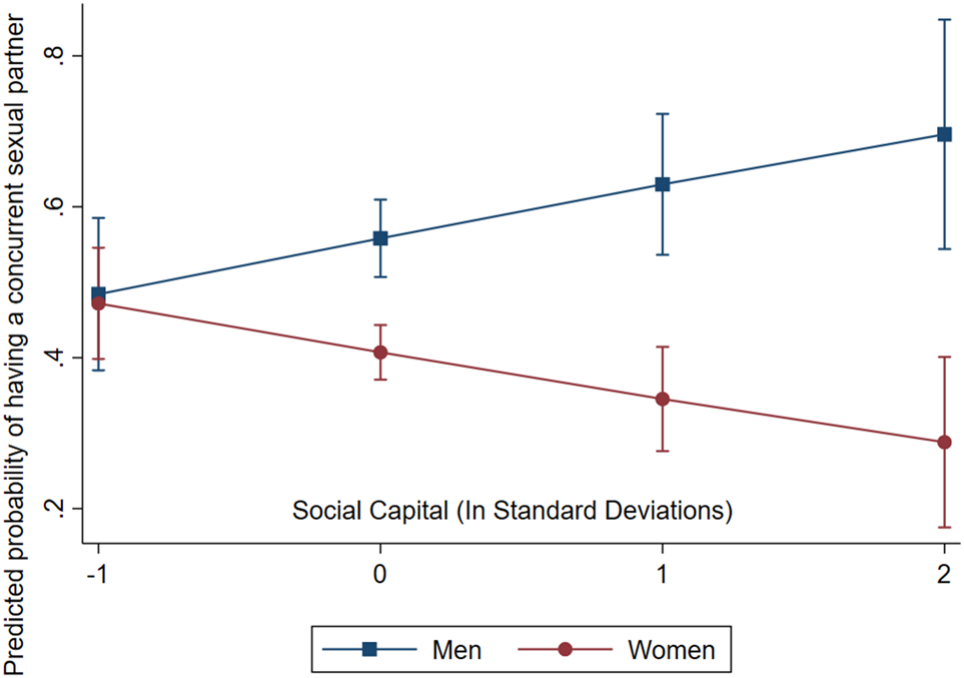
The graphical association showing that the risk of having a concurrent sexual partner in the past year is higher for men compared to women as social capital levels increase

Table 2 shows the multivariable logistic regression models for sexual concurrency in the current year. In initial main effect models, gender but not social capital was significant (result not displayed). From the interaction models, we found that gender significantly modified the association between social capital and sexual concurrency, while adjusting for sociodemographic characteristics (Model 1, *χ*^2^ (df = 1) = 5.02, *p* = 0.025). Specifically, higher social capital was associated with lower odds of sexual concurrency among women [aOR] = 0.60 (95% CI 0.38, 0.94), p = 0.025 compared to men [aOR] = 1.66 (95% CI 1.06, 2.59), p = 0.025. Model 2 contains the addition of psychosocial factors (depressive symptoms and the interaction between AUDIT-c hazardous alcohol use * self-reported HIV risk) in the multivariable model for sexual concurrency in the current year. The gender by social capital effect modification remained significant (Model 1, *χ*^2^ (df = 1) = 4.45, *p* = 0.035). Addition of these variables did not meaningfully alter the *magnitude* of associations for women [aOR] = 0.62 (95% CI 0.39, 0.97), p = 0.035 or men [aOR] = 1.62 (95% CI 1.03, 2.53), p = 0.035. Men’s risk was still about 60% higher compared to women at mean levels of social capital. The graphical illustration in Fig. 1 shows a diverging direction of trends and different magnitudes by gender, which provides compelling evidence that men’s risk of being in a concurrent sexual relationship is higher as social capital increased. Table 3 contains results of analyses from sexual concurrency in the past year. The pattern of results and the interaction plot (not presented) were very similar to those in the current year and so are not presented. The previous effect modification coefficients were presented on the multiplicative scale for men and women. The relative excess risk due to the interaction (RERI), which is effect modification on the additive scale, was − 0.56 95% CI (− 1.09, − 0.03), p = 0.08. This results suggests that if social capital was a scarce resource, then social capital interventions for sexual concurrency might have a greater public health benefit if targeted towards men, and this interpretation was based on us coding men 0 and women 1 in the statistical models.

**Table 2:** Multivariable association between social capital, gender, and sexual concurrency in the current year

Sexual concurrency, current year (^a^N = 1186)	Model 1	Model 2
	aOR (95%CI)	aOR (95%CI)
Social capital	1.27 (0.89, 1.81)^ns^	1.24 (0.86, 1.78)^ns^
Gender (women)	0.52 (0.39, 0.69)^***^	0.52 (0.39, 0.70)^***^
Gender * social capital (men)	1.66 (1.06, 2.59)^*^	1.62 (1.03, 2.53)^*^
Gender * social capital (women)	0.60 (0.38, 0.93)^*^	0.62 (0.39, 0.97)^*^
	^b^(Model 1, *χ*^2^ (df = 1) = 5.02, *p* = .025)	^b^(Model 1, *χ*^2^ (df = 1) = 4.45, *p* = .034)

Model 1 adjusts for age, sexual orientation, marital status, education, income, employment, and on public assistance

Model 2 adjusts for Model 1 + self-reported depression, and a two-way interaction term between self-reported HIV risk and excessive alcohol use using the AUDIT-C binary variable tailored for sex-specific scores

*ns* not significant

^a^Multivariable model sample size

^b^Test for interaction between gender*social capital on the marginal scale that accounts for baseline differences in odds ratios

*p < .05; **p < .01; ***p < .001; p < .10

**Table 3:** Multivariable association between social capital, gender, and sexual concurrency in the past year

Sexual concurrency, past year N^a^ (1186)	Model 1	Model 2
	aOR (95%CI)	aOR (95%CI)
Social capital	1.39 (0.97, 1.99)^+^	1.37 (0.95, 1.97)^+^
Gender (women)	0.53 (0.39, 0.70)***	0.52 (0.39, 0.70)^***^
Gender* Social Capital (men)	1.84 (1.17, 2.87)^**^	1.82 (1.16, 2.87)^**^
Gender * Social Capital (women)	0.54 (0.35, 0.85)**	0.55 (0.35, 0.86)^**^
	^b^(Model 1, *χ*^2^ (df = 1) = 7.13, *p* = .008)	^b^(Model 1, *χ*^2^ (df = 1) = 6.85, *p* = .009)

Model 1 adjusts for age, sexual orientation, marital status, education, income, employment, and on public assistance

Model 2 adjusts for Model 1 + self-reported depression, and a two-way interaction term between self-reported HIV risk and excessive alcohol use using the AUDIT-C binary variable tailored for sex-specific scores

*ns* not significant

^a^Multivariable model sample size

^b^Test for interaction between gender*social capital on the marginal scale that accounts for baseline differences in odds ratios

*p < .05; **p < .01; ***p < .001; + p < .10

## Discussion

Sexual concurrency remains a key risk factor that contributes to high HIV incidence among African Americans, particularly in the south, and more likely to have an adverse impact on women. Social capital has been associated with other self-reported HIV risk behaviors such condom use. However, no prior studies examined its association with sexual concurrency. Therefore, we examined whether social capital was associated with sexual concurrency, and whether gender moderated any association found. Our second question was informed by the theory of gender and power and empirical work on the topic [40, 49, 50].

We found strong statistical evidence for divergent slopes and magnitude in the association between social capital and sexual concurrency, therefore we conclude effect modification based on gender. Specifically, as social capital increased, the predicted probability that one would have a concurrent sexual partner was increasing for men but decreasing for women.

This gender effect modification pattern could potentially be explained by a few possibilities. First, we speculate that social capital is both accumulated and used differentially by men and women. For instance, one study from East Zimbabwe that examined gender, and HIV incidence in association with social capital through indicators of community group membership participation [58, 59]. The authors found that social capital was associated with lower HIV incidence for women compared to men and that gender difference was primarily driven through women’s increased self-efficacy to persuade their partners to practice safer sex [58, 59]. In our study, social capital was measured via a social cohesion approach (i.e., including indicators such as perceived trust and belonging to community). From the social cohesion perspective, it is possible that women with higher social cohesion are protected from some social and structural factors associated with sexual concurrency, such as economic dependence on partners [60]. Women in this possible scenario could be engaged in social networks with positive norms that discourage specific behavioral characteristics [61] and may become more trusting of their community and in turn, the partners found in their community. Women may also feel more able to seek economic help from the community rather than through overlapping sexual partners.

Second, it is possible that gender differences may be a function of where social capital was obtained. In the East Zimbabwe study, men were more likely to participate in sports clubs and political associations, whereas women frequently participated in church groups and events that discussed AIDS [58]. In Jackson, MS, how social capital is generated for men and women may also be different and possibly depend on one’s neighborhood of residence. Social capital has often been conceptualized at the neighborhood level and neighborhood social cohesion has been associated with condom use [62], although ther are no studies linking it to sexual concurrency.

Third, gender differences in the association could be differential participation in social capital generating activities. It is well documented that African American women have higher levels of religious participation compared to men [63] and that the Black Church (i.e., religious and faith congregations with predominantly black parishioners) tend to be more conservative regarding sexual behaviors [64]. Therefore, it is possible that religious involvement may be on a causal pathway through which social capital influenced lower concurrency among women by discouraging risky sexual behaviors such as sexual concurrency [65].

Fourth, divergent gender patterns could possibly be explained by the economic and psychosocial coping opportunities that social capital provides, which may differ for men and women. Some posit that for men, having multiple overlapping sexual partnerships may be a strategy for survival or primary source of social support and capital [66]. Prior research on sexual concurrency among African American men in Philadelphia found that men relied on concurrent sexual partners for food, shelter, and economic support [67]. However, there is another side not often discussed, which are that some men may be seeking psychosocial and emotional support to buffer against hardships and constant threat associated with being a black male in America. This point is well illustrated in barbershop intervention study that assessed HIV prevention among heterosexually-identified men [68]. Men reported in qualitative interviews that multiple overlapping sexual partners provided emptional support and security, as demonstrated by one participant who said the women helped him cope with abandonment he experienced earlier in life [68].

For women, the reasons may be different. One study in Jackson, MS found that women were more likely to engage in sexual concurrency if they had one or more partners with a history of incarceration [51]. One study that used a Black feminist framework analyzed qualitative data posited that some African American women may engage in concurrent sexual partnership as a form of resistance to male-dominated sexual norms within society [69]. However, although autonomy may be important for self-esteem and resilience, studies have shown low or inconsistent use of preventive self-reported HIV risk behaviors among women who engaged in those relationships, which is problematic for HIV prevention [69, 70].

Societal expectations could potentially account for these gender differences we observed. It is possible that women may be socially forced to comply with gender norms of having one partner [71]. Gender norms, when intersecting with race relations in Jackson, MS, have had a lasting negative impact on African American women in this sociocultural conservative region of the country [72]. At the same time, male-dominated gender relations may also make it easier for men to engage in double standards so that the more social capital they have, the more access and opportunity to engage in extramarital affairs and concurrent sexual relationships [73].

A secondary objective of our study was to identify whether psychosocial factors could attenuate any gender differences observed. Previous work by Gregson et al. [49] found that men were more likely than women to participate in events that involved alcohol use. Additionally, excessive alcohol use and self-reported depression may indicate social problems for which seeking social support (whether through sexual partnerships or otherwise) may be a coping mechanism [74, 75]. Adjusting for these factors did not materially alter the degree to which gender modified the relationship between social capital and sexual concurrency in our study.

Our study is perhaps the first published work internationally or domestically to examine the association between social capital and sexual concurrency and demonstrate effect modification by gender. We build upon prior work that highlighted the socio-contextual determinants of self-reported HIV risk among African Americans concerning sexual concurrency as a primary driver [76]. Our findings, if confirmed in replicate studies with other populations, could have major implications for HIV-behavioral and biomedical interventions. Stakeholders will then have to decide who to target for interventions. Based on the RERI coefficient < 0, the statistical model would suggest a greater public health impact by targeting men.

Statistics, though need not be the drivig factor. One could easily argue for intervening among women to keep sexual concurrency low among them by adding social capital components [65] to existing and successful evidence-based interventions to reduce HIV risk among African-American women (e.g., Healthy Love) [77]. In that intervention, one could potentially add components to the module that show women how to identify resources of social capital within their networks and leverage that for strength in negotiating relationships and other sexual behaviors [78]. In his 2015 movie *Chi-Raq*, Spike Lee illustrates how black women gathered together and withheld sex from their husbands [79], which was their intervention to reduce violence in their neighborhood. Although the movie was fiction, women demonstrated several components of social capital [80] such as strong connections to their neighborhoods, which led them to take action in response to a public health problem that threatned people they love.

For men, interventions may be more complex because the HIV epidemiologic profile among black men is patterned by transmission status. Male to male sexual contact among black men accounts for 80% of new HIV diagnosis compared to 14% from heterosexual contact. Therefore, the implications for sexual concurrency will depend on which subgroup of men are targeted. For example, the Barbershop Talk with Brothers (BTWB) targeted heterosexually identified men and is one successful intervention that showed a 60% increase in likelihood of using condoms during sex, among the intervention compared to control group [81]. Earlier work from that same sample of men identified sexual concurrency as an issue, though it was not an end point of the study [68].

Social cohesion and capital are group level properties and so its possible for one to conceptualize barbershops as “neighborhoods” since each shop has a unique culture, social norms and geographic boundaries. Barbershops are an ideal setting for social cohesion generation among black men because there is often perceived trust and belongingness. Therefore, one can potentially add modules to BTWB (and similar interventions) to discuss the sexual, HIV-related and other societal implications of having multiple overlapping sexual partners.

For black men who have sex with men (MSM), there are a number of interventions with varying success, which have foused mainly on sexual risk reduction strategies including: consistent condom use and timely HIV testing, dealing with homophobia, sexual identity, and partner selection, but none on systematically addressed concurrent sexual partnerships [82]. Nevertheless, there are interventions such as Many Men Many Voices (3MV) that are based in neighborhoods and developed and delivered by community-based organziations that serve black MSM [83]. One randomized trial study evaluating 3MV found a 25% greater reduction number of casual sexual partners, among those in the intervention compared to comparison group [83]. Future interventions could leverage prior social capital and HIV-related behavior research among black MSM in the U.S [84–86]. and internationally [87] to develop modules that are consistent with social cohesion indicators.

Our results are subject to several limitations. This sample was drawn from an urban STI clinic with individuals at particularly high risk; thus our findings may not generalize to other African American groups with more variable risk. Our data were from an observational cross-sectional sample, so we cannot make causal inference statements about the relationship between social capital and sexual concurrency. The social capital measures in these data were limited to social cohesion indicators and at the individual level. We did not collect the residential address information from participants, nor did we ask about other social capital indicators. There are alternative and numerous conceptualizations of social capital beyond cognitive measures (e.g., informal social control and civic participation) [17, 26] that may be important for sexual concurrency. Social capital has also been frequently conceived as a property of neighborhoods. Studies that examine different measures and at the neighborhood level may provide further insight into how men and women interact with their community and any subsequent impact on sexual concurrency. Researchers should select those measures and units of analysis informed by specific theories about relationships between social capital and behaviors they study [17, 88].

There are multiple definitions of sexual concurrency (e.g., UNAIDS version, which additionally asks about the intention to have sex again with the partner). We analyzed only self-reported concurrency because of the larger sample of non-missing data and previous work indicating (1) high overlap across definitions, and (2) no significant differences in the sociodemographic correlates of concurrency based on either definition [51]. Self-reported concurrency measures may be influenced social desirability bias that is gendered. Men tend to overreport sexual partners, so the gender differences we found may be overestimated.

Lastly, in this study, we focused on a handful of psychosocial predictors (excessive alcohol use, self-reported depression, and self-reported HIV risk) as potential explanatory mechanisms. There are other structural factors, particularly incarceration [11, 12, 47] likely to significantly explain gender differences in sexual concurrency. However, incarceration, which falls under a greater umbrella of involvement with the criminal legal system is complex because of nuances such as duration and timing of incarceration [89], frequency of arrests, involvement in jails vs prison. A topic with such depth was outside the scope of the current study.

## Conclusion

African Americans compared to other racial and ethnic groups, particularly in the southern cities such as Jackson, MS continue to experience high rates of new HIV infection [4]. Racial disparities in HIV infection are driven primarily through higher HIV prevalence among sexual networks, assortative mixing among African Americans, and engaging in concurrent sexual partnerships, which increases the likelihood of HIV transmission [5, 9]. We show that for men, higher social capital was associated with higher risk of having overlapping sexual partners but lower overlapping partners for women. These findings suggest that in this area, existing behavioral interventions should consider adding social cohesion components to address HIV-related disparities for African Americans. Replicate studies in other southern cities are necessary to support these findings and identify which types of social capital components are best to use.
